# Collembola at three alpine subarctic sites resistant to twenty years of experimental warming

**DOI:** 10.1038/srep18161

**Published:** 2015-12-16

**Authors:** Juha M. Alatalo, Annika K. Jägerbrand, Peter Čuchta

**Affiliations:** 1 Department of Biological and Environmental Sciences, College of Arts and Sciences, Qatar University, P.O. Box 2713, Doha, Qatar; 2 VTI, Swedish National Road and Transport Research Institute, Box 55685, 102 15 Stockholm, Sweden; 3 Biology Centre, Institute of Soil Biology, Academy of Science of the Czech Republic, 370 05 České Budějovice, Czech Republic

## Abstract

This study examined the effects of micro-scale, site and 19 and 21 years of experimental warming on Collembola in three contrasting alpine subarctic plant communities (poor heath, rich meadow, wet meadow). Unexpectedly, experimental long-term warming had no significant effect on species richness, effective number of species, total abundance or abundance of any Collembola species. There were micro-scale effects on species richness, total abundance, and abundance of 10 of 35 species identified. Site had significant effect on effective number of species, and abundance of six species, with abundance patterns differing between sites. Site and long-term warming gave non-significant trends in species richness. The highest species richness was observed in poor heath, but mean species richness tended to be highest in rich meadow and lowest in wet meadow. Warming showed a tendency for a negative impact on species richness. This long-term warming experiment across three contrasting sites revealed that Collembola is capable of high resistance to climate change. We demonstrated that micro-scale and site effects are the main controlling factors for Collembola abundance in high alpine subarctic environments. Thus local heterogeneity is likely important for soil fauna composition and may play a crucial role in buffering Collembola against future climate change.

Climate change is predicted to show a relatively fast pace of increase in polar regions, forcing their ecosystems to respond rapidly to change^1^,^2^. Although a number of studies have shown recent poleward movement of organisms, this cannot always be linked to climate change^3^. Changes in vascular plant communities have already been observed on species, functional and community level. For example, warming in the Arctic has been shown to increase plant growth and cause shifts in vegetation composition, with shrubs increasing in abundance^4^,^5^,^6^,^7^.

However, the majority of experimental studies simulating climate change in alpine and polar regions have focused on plants, especially vascular plants^8^, while studies on bryophytes and lichens at species level are rare^9^, and studies on soil fauna are even rarer^10^. There is a need to incorporate the responses of below-ground arthropod communities in climate change simulations, as they play an important role in ecosystem functioning^11^,^12^.

In fact, arthropods are among the most dominant groups of organisms in term of species richness and abundance and play a crucial role in all terrestrial ecosystems^13^. As many soil arthropods in polar regions have traits that allow them to remain dormant through the cold winter and become active during the more favourable summer conditions, it has been argued that they may be at risk of exposure to more competitive species if climate change results in a warmer environment where more species can thrive. Thus climate change may cause changes in the soil communities, with implications for ecosystem functioning^14^,^15^,^16^.

The few experimental warming studies conducted to date in polar regions show contrasting responses to warming^10^. For example, Collembola have been reported to respond negatively^17^,^18^,^19^,^20^,^21^,^22^,^23^, positively^19^,^22^ or neutrally^18^,^20^. Neutral responses to experimental warming have also been reported in studies of non-polar regions^24^. As open-top chambers (OTCs) can cause a decline in soil moisture^23^,^25^,^26^, and as Collembola are thought to be sensitive to low soil moisture levels, they have been found to be negatively affected in OTC warming experiments on ecosystems where soil moisture is a limiting factor^10^,^17^. The importance of soil moisture has been demonstrated in experiments using infrared heating in an alpine meadow, which had a negative effect on mesofauna biomass and diversity in drier parts and a positive effect in moist sub-areas^27^.

In Arctic soils, Collembola have also been shown to display large micro-scale variations in their distribution, caused by the plant species present^28^. The importance of plant diversity for soil food webs has also been shown in a 13-year grassland experiment^29^.

The majority of the few experimental climate change studies that exist on Collembola in polar and alpine regions are short-term^17^,^19^,^20^,^21^,^22^, but there is a need for long-term data in order to predict the long-term consequences of climate change. To the best of our knowledge, there are only two published long-term experimental climate change studies, one with 11 years of warming in a fell field and 12 years in a heath^18^, and one with 16 years of warming in a mountain birch forest^23^.

Here we report the responses of Collembola in alpine subarctic Sweden to 19 years of warming in a rich meadow and a poor heath community and to 21 years of warming in a wet meadow community. We studied in particular the potential effects of micro-scale, site and experimental long-term warming. The spatial aspect is important because the study sites are highly heterogeneous regarding plant distribution and has a thin heterogeneous soil layer. We examined whether this was reflected in below-ground Collembola communities, as has been shown in the high Arctic Svalbard^28^.

To our knowledge, the present study represents the longest experimental climate change study on Collembola reported to date. The specific hypothesis tested was that long-term warming has significant negative impact on Collembolan abundance and species richness.

## Results

### Impact on species richness, diversity and evenness

**Figure 1:**
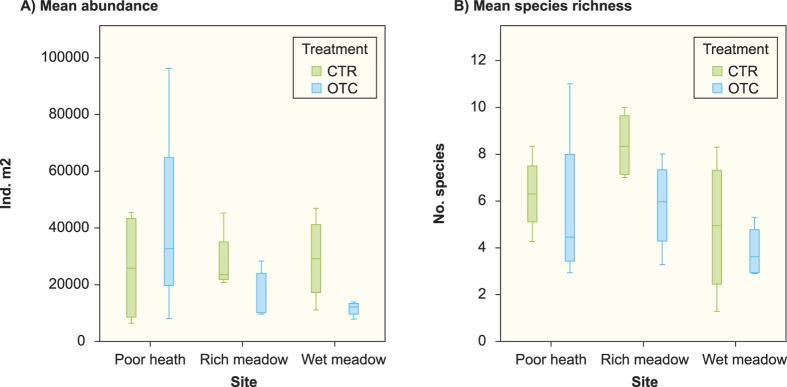
Box plots of mean total abundance (ind. m^2^) and mean number of Collembola species at the poor heath, rich meadow, wet meadow sites and in the control (CTR) and warming treatment (OTC) at Latnjajaure field station, subarctic Sweden. (**A**) Mean total abundance among sites and treatments (**B**) mean species richness among sites and treatments. Boxplots show the 10^th^ – 90^th^ percentiles of the data; n = 4 for each site and treatment.

In total, we recorded 35 species of Collembola at the three sites (Table 1), 25 species in the poor heath, 24 species in the rich meadow and 21 species in the wet meadow. Micro-scale had a significant effect (*P* = 0.002) on species richness, while site (*P* = 0.086) and experimental long-term warming (*P* = 0.101) had non-significant effects. However, the experimental long-term warming tended to have a negative effect on species richness at all sites (Fig. 1). Average species richness decreased from 6.3 to 5.8 in the poor heath, from 8.4 to 5.8 in the rich meadow and from 4.9 to 3.9 in the wet meadow. The rich meadow tended to have the highest mean species richness, followed by the poor heath, while the wet meadow had the lowest species richness (Fig. 1). The effective number of species (exponential of Shannon’s entropy) differed significantly among sites (*P* = 0.02), the results following the same pattern as species richness with the rich meadow having the highest effective number of species, and the wet meadow the lowest effective number of species (Table 2). There was a non-significant tendency for a site effect on Pielou’s evenness index (*P* = 0.068), with the rich meadow tending to have higher evenness (Table 2). Long-term warming had no significant effect on effective number of species (*P* = 0.347), or Pielou’s evenness index (*P* = 0.75).

### Impact on total Collembola abundance

As regards total Collembola abundance, we only found significant effects for micro-scale (*P* = 0.006), while site (*P* = 0.816) and experimental long-term warming (*P* = 0.198) had no significant effect (Fig. 1). Total abundance tended to be higher in the poor heath than in the rich meadow and the wet meadow (Fig. 1).

### Species specific responses

**Figure 2:**
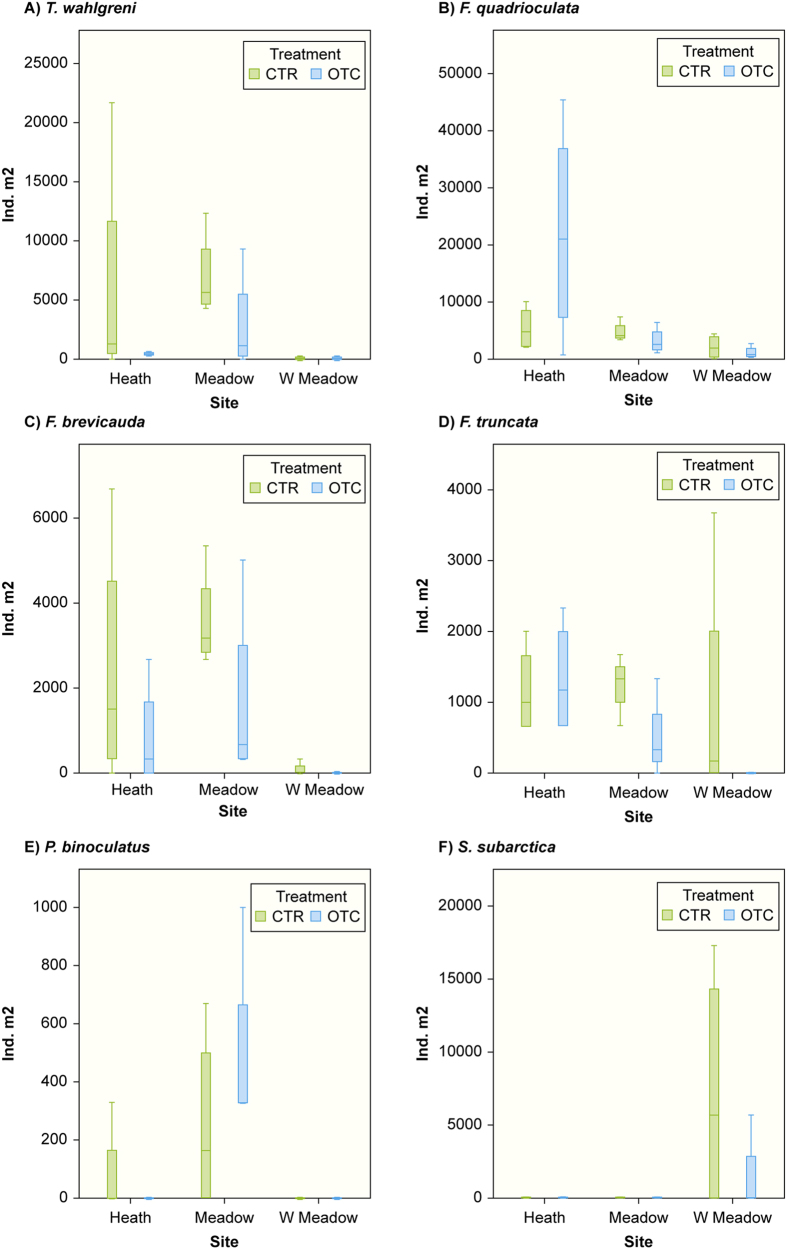
Box plots of mean abundance (ind. m^2^) of six Collembola species at the poor heath, rich meadow, wet meadow sites and in the control (CTR) and warming treatment (OTC) at Latnjajaure field station, subarctic Sweden. (**A**) *Tetracanthella wahlgreni,* (**B**) *Folsomia quadrioculata,* (**C**) *F. brevicauda,* (**D**) *Friesea truncata,* (**E**) *Pseudanurophorus binoculatus* and (**F**) *Scutisotoma subarctica.* Boxplots show the 10^th^–90^th^ percentiles of the data; n = 4 for each site and treatment.

On species level both micro-scale and site had significant effects on species abundance. We found significant micro-scale effects on the abundance of 10 Collembola species: *Tetracanthella wahlgreni* (*P* = 0.017), *F. quadrioculata* (*P* = 0.001), *F. brevicauda* (*P* = 0.001), *F. palaearctica* (*P* = 0.008), *I. minor* (*P* = 0.002), *P. notabilis* (*P* = 0.025), *Desoria neglecta* (*P* = 0.08), *D. olivacea* (*P* = 0.022), *Protaphorura pseudovanderdrifti* (*P* = 0.043) and *Scutisotoma subarctica* (*P* < 0.001). There was a significant effect of site on the abundance of six species: *T. wahlgreni* (*P* = 0.003)*, F. quadrioculata* (*P* = 0.024)*, F. brevicauda* (*P* = 0.002)*, Friesea truncata* (*P* = 0.017)*, Pseudanurophorus binoculatus* (*P* = 0.002), and *Scutisotoma subarctica* (*P* = 0.01), with the different species having contrasting abundance among sites (Fig. 2), and non-significant effects on *Lepidocyrtus lignorum* (*P* = 0.08), *Tullbergia arctica* (*P* = 0.12), and *Willemia denisi* (*P* = 0.12). Experimental long-term warming had no significant effect on any species.

**Figure 3:**
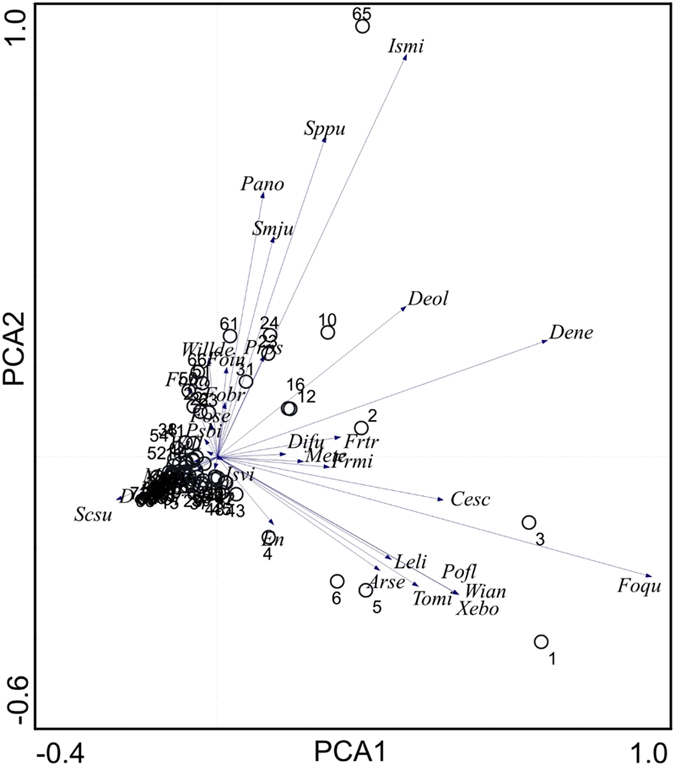
Plot of principal component analysis (PCA) on species abundances of collembola. PCA 1 and 2 explains 69.7% of the variation (48.5% and 21.2%, respectively). For species abbreviations see Supplementary raw data.

The principal component analysis (PCA) explained a large percentage of the total variation of species occurrences, for the first two axes a total of 69.7% (Fig. 3). A few species tended to dominate in both axes, for PCA1 in the right directions, *Folsomia quadrioculata* (Foqu), *Willemia anophthalma* (Wian), *Xenylla boerneri* (Xebo) and *Pogonognathellus flavescens* (Pofl) had high dominance in the OTC plots (number 1,3,4,5,6) in the heath community. PCA2 was dominated by *Isotomiella minor* (Ismi), *Sphaeridia pumilis* (Sppu), and *Parisotoma notabilis* (Pano) (Fig. 3), mainly explained by high occurrence in plot 65, a control plot at the wet meadow site. Redundancy analysis (RDA) showed that only 2.9% of the variation (see eigenvalues in Table 3) could be explained by the constraining factors (site, treatment), indicating that other factors than site and treatment was more important in explaining the community structure and variance of Collembola.

## Discussion

In terms of total species richness, we expected to find the lowest species richness in the poor heath, as that site was the driest of the three sites included in the study and had a more sparse vegetation cover. This as Collembola abundance is often correlated with moisture availability, as desiccation is a strong limitation to many species^21^. Therefore we were surprised to find that total species richness was highest in the poor heath. However, both the mean species richness and the effective number of species was highest in the rich meadow. This shows that Collembola can thrive even in dry sites with sparse vegetation cover. It also indicates that Collembola communities may possess high resistance to climate change.

We had expected a decline in total Collembola abundance in response to long-term warming, as OTCs have frequently been shown to decrease soil moisture as a side-effect^23^,^25^,^26^. However, our results are similar to those found in alpine Norway after four years of experimental warming, where the Collembola community was found to be largely resistant to the warming perturbation. Only three low-frequency species were significantly affected by the warming in that study, two (*Micranurida forsslundi* and *Isotoma* sp.) which increased in abundance and one (*T. wahlgreni*) which decreased^22^. In Abisko, *i.e*. near our high alpine experimental sites at Latnjavagge, two studies on experimental long-term warming have reported contrasting results for different field sites (plant communities). One study found that 16 years of warming caused a significant decline in Collembola abundance (by 51%) and in species richness (from an average of 14 to 12) in a mountain birch forest at 400 m a.s.l.^23^. The other study^18^ found that 5 years of experimental warming in a glade (open space in the forest) and 11 years of warming in a fell field (low growing vegetation among rocks and bare ground) decreased abundance of Collembola, whereas 12 years of warming in a heath (vegetation dominated by dwarf shrubs) had no strong effects. The responses in the glade were also inconsistent between years. A study comparing short-term responses of Collembola in High Arctic Svalbard and sub-arctic Sweden found contrasting responses between the sparsely vegetated polar semidesert (negative effect) and the densely vegetated subarctic heath (no effect). This was thought to be caused by faster drying of the soil by OTCs in the semidesert, causing desiccation of Collembola^30^. Furthermore, a short-term experiment in a grassland reported an increase in the abundance of Collembola to warming with OTCs^31^. Thus, the responses appear to be highly site-specific, sometimes even within short distances. Indeed, our study found that site had a significant effect on the abundance of seven Collembola species, while experimental warming did not affect any species significantly. This is in line with previous findings that Collembola abundance can vary significantly on micro-scale^28^.

In our study the factors that were most important for Collembola communities were micro-scale effect, followed by site effect, which emphasises the importance of small-scale spatial heterogeneity for soil fauna. With Collembola exhibiting this high spatial variance, a much larger number of replicates is most likely needed in order to detect potential treatment effects, as the natural heterogeneity of Collembola distribution may mask the effect of warming. In fact, on discovering the great heterogeneity at the site we have established a new long-term warming experiment in one plant community with the sole purpose of studying the impact on soil fauna. With 20 plots for each treatment, we hope this will provide better knowledge of the effects over time and higher statistical power.

The responses of microarthropods have been suggested to be controlled in a bottom-up manner, with changes in food availability being more important than direct climatic influences^18^. Our results tended to support this and it could explain why micro-scale and site were more important than experimental long-term warming.

Another plausible explanation for the site effect could be a natural nutrient and soil moisture gradient between sites. We did not measure nutrient content or moisture level here, but this has been done in a previous study in the poor heath and rich meadow. It showed that the poor heath is a more nutrient-limited ecosystem with lower soil moisture, shallower organic soil horizon and three-fold lower N mineralisation rate than meadow ecosystems (mesic meadow and meadow snowbed)^32^. Therefore it is likely that the three sites constituted a soil moisture gradient from low (poor heath) to high soil moisture level (wet meadow), with the rich meadow being intermediate. This natural soil moisture gradient may partly explain the differences in abundance and species richness found between sites.

Our experiment did not take into account the potential arrival of new species from warmer latitudes, but only determined changes in species already present. However, terrestrial Collembola have been shown to be capable of long-range dispersal by birds, water and air^33^,^34^,^35^ and arrival of new species might change the community structures in the future due to increased competition.

Previous climate change studies have mainly focused on the potential impact on plant communities, but to better understand the potential impact on ecosystems long-term studies are needed on belowground biota. These have an important function, as soil fauna control many soil processes and are linked with the above-ground vegetation^16^. The role of microarthropods is important in polar ecosystems, as larger soil fauna are less abundant^36^.

Contrary to our hypothesis that predicted a negative effect from long-term warming, our experiment across three contrasting sites suggest that Collembola may display high resistance to climate change, since total abundance, individual species abundances and species richness were not significantly affected by decades of experimental warming. Micro-scale and site effects seem to be the main controlling factors for Collembola abundance at the high alpine subarctic environment studied. Thus local heterogeneity is likely very important for soil fauna composition and may play a crucial role in buffering Collembola against future climate change.

## Methods

### Study area

Three experimental sites were selected at Latnjajaure Field Station (LFS) in northern Sweden, at 1000 m elevation in the Latnjavagge valley (68°21´N, 18°29´E) near Abisko. The climate in the area can be classified as sub-arctic, with cool summers, relatively mild snow-rich winters, and a snow cover in the valley for most of the year. Mean annual temperature ranges from −1 to −3 °C and total annual precipitation ranges between 600 and 1100 mm. The valley is highly diverse as regards physical conditions, ranging from dry to wet and from poor and acidic to base-rich, variations reflected in its plant communities^8^,^37^.

### Experimental design

Warming was induced using OTCs, which increase the temperature by 1.5–3 °C compared with control plots with ambient temperature^37^. In the wet meadow community, 10 plots with homogeneous vegetation cover were chosen in 1993 and half of these were assigned to OTCs and half to controls in a pairwise design. In this study we sampled four OTC plots and their control plots in the wet meadow to have equal sample size to the rich meadow and poor heath. In both the rich meadow and heath community, eight plots (1 m x 1 m) were randomly assigned to treatments in 1995, half to OTCs and half to controls. The OTCs were left on plots with warming treatments year-around at all three sites, but the warming only occurs during the short snow free period. The warming effect of OTCs being related with irradiance^38^. As the plots are part of long-term experiments on plant communities, we tried to avoid large-scale destructive sampling that would ruin them for future research. By the sampling year (2013), the wet meadow had experienced 21 years of experimental warming and the rich meadow and poor heath 19 years of experimental warming. Detailed information about the plant communities can be found in previous papers^37^,^39^.

### Collembola sampling and analyses

In early August 2013, we extracted three soil cores (randomly within each plot) from four plots with OTCs and four control plots in each of the three plant communities. These samples from the upper soil layer with roots, represented soil cores of 3.6 cm diameter (10 cm^2^ cross-sectional area) to a maximum depth of 7–12 cm (depending on soil layer thickness). The samples were stored in plastic bags in cool-boxes until extraction, which was carried out within 5 days of sampling in the field. The material was extracted in a modified high-gradient extraction apparatus for 7 days at the Czech Academy of Sciences, Ceske Budovice^40^. Collembola were identified to species and family level using basic taxonomic keys for Symphypleona^41^, Entomobryomorpha^42^, Poduromorpha^43^, Hypogastruridae^44^, Isotomidae^45^, Tullbergiinae^46^, and class Collembola^47^. Community parameters were calculated for comparison of the collembolan assemblages: abundance (A), species richness (S), effective number of species (expH = exponential of Shannon diversity) which is the number of equally abundant species needed for the average proportional abundance of the species to equal that observed in the dataset (where all species may not be equally abundant)^48^, and Pielou index of evenness (J′) (Table 2).

### Statistical analyses

As the data did not meet normal assumptions after transformation we used the conservative nonparametric Mann-Whitney U test to analyse the effect of treatment on total Collembola density, species richness, effective number of species (expH), Pielou’s evenness index (J’), and density of individual species across all plant communities. We used the Kruskal-Wallis test for analysing the effect of site and micro-scale (plot scale) on total Collembola density, species richness, effective number of species (expH), Pielou’s evenness index (J’), and density of individual species. All non-parametric statistical analyses were performed with SPSS version 21 (IBM) for Macintosh. We pooled the data within plots for the analyses of treatment and site effects, but not micro-scale effects. We are aware of the potential problem of pseudoreplication for the non-pooled data. The reason for including micro-scale effects is that others have shown that Collembolan abundance in polar regions can be heavily influenced by micro-scale heterogeneity^28^.

Community structure was analysed by detrended correspondence analysis (DCA) based on species occurrences with standard default settings. DCA showed that the data were homogenous in species composition since the axes were of intermediate lengths, between 3 and 4 standard deviation units for species turnover, indicating that either linear or unimodal techniques would be appropriate to apply. We conducted both linear and unimodal variants of unconstrained (principal component analysis, PCA and correspondence analysis, CA) and constrained (redundancy analysis, RDA and canonical correspondence analysis, CCA) multivariate analysis to evaluate which technique would be most optimal for extraction of the variation in the community data. The cumulative percentage explained variance from the unimodal multivariate analysis techniques yielded both a lower fit and we therefore only report the results from the linear analysis. The constrained multivariate analysis (in this case RDA) incorporate environmental or other constraining factors to optimize the fit of the data and evaluate the significance of treatments (i.e. control, OTC) and site (i.e. heath, meadow and wet meadow) by Monte Carlo permutation tests (1,000 permutations on non-transformed data). PCA was performed with no transformations, centering on species-correlations and default settings. Multivariate analysis were performed in CANOCO 4.5^49^.

## Additional Information

**How to cite this article**: Alatalo, J. M. *et al.* Collembola at three alpine subarctic sites resistant to twenty years of experimental warming. *Sci. Rep.* **5**, 18161; doi: 10.1038/srep18161 (2015).

## Supplementary Material

Supplementary Information

## Figures and Tables

**Table 1:** List of Collembola species found in the poor heath (H), rich meadow (R) and wet meadow (W) plant communities at Latnjajaure Field Station, northern Sweden.

Order **Poduromorpha**	Order **Entomobryomorpha**	Superfamily **Tomoceroidea**
		Family **Tomoceridae:**
Superfamily **Hypogastruroidea**	Superfamily **Entomobryoidea**	*Pogonognathellus flavescens* (H)
Family **Hypogastruridae:**	Family **Entomobryidae:**	*Tomocerina minuta* (H, R, W)
*Ceratophysella scotica* (H, R)	*Lepidocyrtus lignorum* (H, R)	
*Willemia anophthalma* (W)	*Pseudosinella* cf. *horaki* (W)	Order **Neelipleona**
*Willemia denisi* (W)	*Willowsia* cf. *buski* (H, R)	
*Xenylla boerneri* (H)	Entomobryidae juv. (H)	Family **Neelidae:**
*Xenylla* cf. *mediterranea* (H)		*Megalothorax minimus* (H, R, W)
	Superfamily **Isotomoidea**	
Superfamily **Neanuroidea**	Family **Isotomidae:**	Order **Symphypleona**
Family **Neanuridae:**	*Desoria neglecta* (H, R, W)	
*Friesea mirabilis* (H, R, W)	*Desoria olivacea* (H, R, W)	Superfamily **Dicyrtomoidea**
*Friesea truncata* (H, R, W)	*Desoria violacea* (H, R, W)	Family **Dicyrtomidae:**
*Micranurida pygmaea* (H)	*Folsomia brevicauda* (H, R, W)	*Dicyrtoma fusca* (H, R)
	*Folsomia* cf. *inoculata* (W)	
Superfamily **Onychiuroidea**	*Folsomia palaearctica* (H, R, W)	Superfamily **Katiannoidea**
Family **Onychiuridae:**	*Folsomia quadrioculata* (H, R, W)	Family **Arrhopalitidae:**
*Protaphorura pseudovanderdrifti* (H, R, W)	*Folsomia sensibilis* (W)	*Arrhopalites secundarius* (H, R)
	*Isotoma viridis* (H, R, W)	
Family **Tullbergiidae:**	*Isotomiella minor* (R, W)	Superfamily **Sminthuridoidea**
*Mesaphorura tenuisensillata* (R)	*Parisotoma notabilis* (H, R, W)	Family **Sminthurididae:**
*Tullbergia arctica* (H, R)	*Pseudanurophorus binoculatus* (R)	*Sminthurides* sp. juv. (R)
	*Scutisotoma subarctica* (W)	*Sphaeridia pumilis* (W)
	*Tetracanthella wahlgreni* (H, R, W)	

**Table 2:** Mean effective number of species and evenness of Collembola in a poor heath, rich meadow and wet meadow after 19 (heath and rich meadow) and 21 years (wet meadow) of experimental warming.

	Heath		Meadow		Wet Meadow	
	OTC	control	OTC	control	OTC	control
expH ± SD	4.95 ± 2.94	7.15 ± 2.55	7.38 ± 1.13	8.41 ± 1.64	4.16 ± 1.56	4.25 ± 3.134
J’ ± SD	0.58 ± 0.25	0.79 ± 0.12	0.83 ± 0.07	0.82 ± 0.06	0.71 ± 0.10	0.54 ± 0.27

expH – effective number of species (exponential of Shannon’s entropy), **J’** – Pielou’s evenness index; n = 4 for each site and treatment, SD = standard deviations).

**Table 3:** Results from redundancy analysis (RDA).

Factor	Axis 1	Axis 2	Axis 3	Axis 4	Total
Eigenvalues	0.029	0.470	0.205	0.078	1.000
Species-environment correlations	0.300	0.000	0.000	0.000	0.300
Cumulative percentage variance					
Of species data	2.9	49.9	70.4	78.2	
Of species-environment relation	100.0	0.0	0.0	0.0	
